# Association between frailty and physical performance in older patients with heart failure

**DOI:** 10.1002/clc.24142

**Published:** 2023-09-07

**Authors:** Uram Jin, Minjae Yoon, Jaehyung Ha, Seung‐Hyun Lee, Doeun Yun, Ji‐Su Kim, Jaewon Oh, Sungha Park, Sang‐Hak Lee, Seok‐Min Kang, Chan Joo Lee

**Affiliations:** ^1^ Department of Cardiology Ajou University School of Medicine Suwon‐si Republic of Korea; ^2^ Division of Cardiology Severance Cardiovascular Hospital and Cardiovascular Research Institute, Yonsei University College of Medicine Seoul Republic of Korea

**Keywords:** frailty, heart failure, cardiopulmonary exercise test, 6‐minute walk test

## Abstract

**Background:**

Frailty is an issue in patients with heart failure (HF). A Korean version of the frailty scale (K‐FRAIL) has been developed.

**Hypothesis:**

We aimed to analyze the relationship between the K‐FRAIL scale and physical performance in patients with HF.

**Methods:**

This study included 142 patients with HF aged ≥65 years from a single center. Muscular fitness was assessed using the handgrip test and knee extensor strength measurement. Aerobic capacity was assessed using the cardiopulmonary exercise test and 6‐min walk test (6MWT). Frailty was assessed using the K‐FRAIL questionnaire.

**Results:**

Peak VO_2_ and 6MWT scores significantly decreased as frailty worsened, but handgrip and knee extensor strength did not. In the multivariate analysis, peak VO_2_ (*β* = −.31; *p* = .002) and 6MWT score (*β* = −.38; *p* < .001) showed significant inverse associations with the K‐FRAIL score. Based on the receiver operating characteristic curve analysis, the cut‐off values of peak VO_2_ (hazard ratio, 5.08; *p* = .023) and 6MWT (hazard ratio, 3.99; *p* = .020) were independent predictors of frailty.

**Conclusion:**

In older patients with HF, physical performance correlates with the degree of frailty. The K‐FRAIL scale is correlated with the peak VO_2_ and 6MWT.

## Graphical abstract

The K‐FRAIL scale is correlated with the peak VO_2_ by cardiopulmonary exercise test and distance walked by 6‐min walk test.

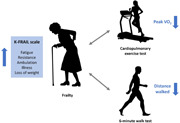

## 1 INTRODUCTION

Frailty is a multidimensional physiological syndrome characterized by an exaggerated decline in the function and reserve of multiple physiological systems to resist stressors.^1^ Apart from the physiological deterioration of the body that occurs with aging, a biological condition in which susceptibility and vulnerability to adverse outcomes is increased can be regarded as frailty.^2^ Frailty can be a strong predictor of adverse clinical outcomes such as death and hospitalization.^3^

Frailty is known to be associated with heart failure (HF), which frequently occurs in older patients. Frailty is often present in HF patients, and it has been reported that 56%–75% of patients hospitalized for HF have frailty.^4^ Conversely, a significant number of patients with frailty complain of symptoms of HF.^5^ This complex interrelationship is presumed to be due to the shared pathophysiology of HF and frailty.^6^ Systemic conditions in patients with HF, such as multiple comorbidities and nutrition deficiency, may be related to the etiology of frailty.^6^ Contrary, chronic inflammation or muscle wasting, considered major pathogenic factors of frailty, can lead to vascular dysfunction, and decreased myocardial reserve, which may contribute to the development of HF.^6^

In patients with HF, frailty worsens not only the quality of life but also the clinical prognosis. Frailty has been proven to be an independent predictor of HF prognosis in community‐ and hospital‐based cohorts.^7^ Therefore, accurate evaluation of the presence and severity of frailty in HF patients is important to individualize treatment.^8^, ^9^ In line with this trend, the need for mandatory frailty assessment in HF patients has been actively discussed, and the Heart Failure Association of the European Society of Cardiology has developed an HF‐specific frailty assessment tool. However, because frailty is a multidimensional dynamic state that includes physical, mental, functional, and social aspects, an objective evaluation is challenging. Evaluation tools that have been developed to accurately evaluate frailty are too numerous and extensive to be used in daily care. Hence, it is necessary to standardize and to simplify the method to evaluate frailty. In Korea, the K‐FRAIL scale, which is a screening tool for frailty status, uses a 5‐item questionnaire, and it has been validated in several studies.^10^, ^11^ The purpose of this study was to determine the prevalence of frailty measured by the K‐FRAIL scale in ambulatory HF patients ≥65 years and to evaluate the usefulness of the K‐FRAIL scale in the assessment of physical fitness according to the degree of frailty.

## 2 MATERIAL AND METHODS

### 2.1 Study participants

We conducted a frailty questionnaire, physical performance assessment, and body composition analysis in HF patients as part of HF management. This study retrospectively analyzed 143 HF patients aged ≥65 years among 262 HF patients who were under stable outpatient follow‐up for more than 1 month and underwent these tests for HF from July 2020 to July 2021. The diagnosis and stages of HF adhered to the criteria of the American College of Cardiology Foundation/American Heart Association.^12^ This study was performed in accordance with the principles of the Declaration of Helsinki (7th revision, 2013). The study protocol was reviewed and approved by the Institutional Review Board of the hospital (4‐2022‐0180), which waived the need for informed consent due to the retrospective nature of the study.

### 2.2 Frailty assessments

**Figure 1:**
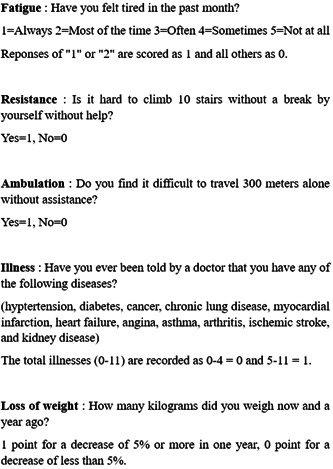
Frailty assessments. The Korean version of the fatigue, resistance, ambulation, illnesses, and loss of weight (K‐FRAIL) scale.

We used the K‐FRAIL scale, which is Korean translated version of FRAIL scale items in African American Health and is a 5‐item questionnaire on fatigue, resistance, ambulation, illness, and weight loss.^10^, ^13^ (Figure 1 and Supporting Information: Figure 1). Fatigue was assessed by asking patients how much time during the past month they felt tired. Resistance was measured by asking patients if they had any difficulty walking up 10 steps alone without resting and without aids. Ambulation was evaluated by asking if they had any difficulty walking 300 m by themselves and without aids. Illness was scored by asking if they had even been told by doctors that they had any of the 11 total diseases. Loss of weight was assessed by asking if they had a weight decline of 5% or greater within the past 12 months. We considered K‐FRAIL scale scores of 0, 1–2, and 3–5 as robust, prefrail, and frail, respectively.

### 2.3 Assessment of aerobic exercise capacity

Functional exercise capacity was evaluated during the maximal treadmill exercise test using the Bruce RAMP protocol with the cardiopulmonary exercise test (CPET) system CASE T2100 (GE Healthcare) under the supervision of a cardiologist. Respiratory gas exchange analysis was performed throughout the exercise protocol using a quark gas analysis system (COSMED). Peak VO_2_ was operationally defined as the highest VO_2_ for a given 15‐ or 20‐s interval within the last 90 s of exercise or the first 30 s of recovery.^14^

The 6‐min walk test (6MWT) was performed according to standard guidelines; however, an 8 m × 8 m square‐shaped continuous track within the cardiac wellness center was used.^15^ A single walk test was administered without practice. Participants were instructed to walk continuously on a track and cover as much ground as possible within 6 min.

### 2.4 Assessment of muscular fitness

A digital grip strength dynamometer (TTK‐5401; Takei Ltd.) was used to measure handgrip strength as an index of upper limb muscle strength.^16^ Three measurements were made on the dominant hand with the elbow extended or flexed while observing for the possibility of a Valsalva effect. We then calculated the average of three measurements.

Two muscular fitness parameters for lower limb muscle strength were assessed using Primus RS, version 11 (Baltimore Therapeutic Equipment Technology), as previously described.^17^ Briefly, maximal voluntary isometric contraction (MVIC) and muscle power (MP) were measured to evaluate the knee extensor muscle strength. The mean value of the top five results from 10 measurements was used in the statistical analysis.

### 2.5 Anthropometric measurements and bioelectrical impedance analysis (BIA)

Anthropometric measurements (body mass index [BMI] and body weight) were obtained from all patients. BIA was used to establish parameters reflecting nutritional status and body composition, including soft lean mass (kg), fat‐free mass (kg), skeletal muscle mass (kg), body fat mass (kg), percent body fat (%), and visceral fat area (cm^2^). BIA measurements were performed using an InBody 720 instrument (InBody), which is a multifrequency bioimpedance device. All parameters were obtained while the patients were standing.

### 2.6 Statistical analysis

Categorical variables are expressed as numbers or percentages, and continuous variables are presented as mean ± standard deviation. Patients were grouped according to the frailty category based on the K‐FRAIL frailty scale system. For group comparison of continuous variables, the Student's *t*‐test, one‐way analysis of variance, Mann–Whitney *U* test, or Kruskal–Wallis test was used, where appropriate. Categorical variables were evaluated using the Chi‐squared test or Fisher's exact test. Associations between frailty status and clinical parameters were evaluated using univariate and multivariate logistic regression analyses. Receiver operating characteristic (ROC) analysis was performed to identify the cut‐off values of peak oxygen uptake (VO_2_), 6MWT, MVIC, MP, and handgrip strength for frailty. The association of the cut‐off values for aerobic exercise capacity and muscular fitness with frailty were evaluated using a multivariable Cox proportional hazard model with adjustments for age, sex, hemoglobin level, estimated glomerular filtration rate (eGFR), and left ventricular ejection fraction (LVEF). All statistical analyses were performed using the SPSS ver. 18 for Windows (SPSS Inc.). Statistical significance was set at *p* < .05.

## 3 RESULTS

### 3.1 Baseline characteristics of patients

Among the 143 patients, 37, 75, and 31 were classified as robust, prefrail, and frail, respectively, according to the K‐FRAIL scale. Table 1 presents the patients' baseline characteristics. The mean ages of robust, prefrail, and frail patients were 72.5, 73.5, and 76.3 years, respectively. Frail patients were older and had a higher BMI, a higher rate of hypertension, and a lower rate of old myocardial infarction (MI) than robust or prefrail patients. Regarding laboratory findings, frail patients had lower hemoglobin levels, lower eGFR, and higher N‐terminal pro‐b‐type natriuretic peptide (NT‐pro‐BNP) levels. There was no difference in the proportion of HF with reduced ejection fraction (HFrEF), LVEF, or echocardiographic measurements related to left ventricular remodeling between the groups.

**Table 1:** Baseline characteristics.

	Robust (*n* = 37)	Prefrail (*n* = 75)	Frail (*n* = 31)	*p*‐Value
Demographic findings				
Age, year	72.5 ± 5.3	73.5 ± 5.4	76.3 ± 6.0*	.015
Men, *n* (%)	18 (48.6%)	35 (46.7%)	11 (35.5%)	.493
Height (cm)	159.6 ± 8.1	159.1 ± 9.3	159.7 ± 8.1	.932
Weight (kg)	63.0 ± 10.6	61.4 ± 8.5	67.3 ± 16.3	.054
BMI (kg/m^2^)	24.7 ± 3.3	24.3 ± 2.9	26.2 ± 5.2*	.048
Hypertension *n* (%)	17 (45.9%)	33 (44.0%)	23 (74.2%)^a^	.014
Diabetes, *n* (%)	4 (10.8%)	24 (32.0%)	9 (29.0%)	.050
CAOD, *n* (%)	5 (13.5%)	23 (30.7%)	11 (35.5%)	.081
PCI, *n* (%)	3 (8.1%)	16 (21.3%)	7 (22.6%)	.180
CABG, *n* (%)	0 (0.0%)	5 (6.7%)	1 (3.2%)	.299
MI, *n* (%)	3 (8.1%)	13 (17.3%)	0 (0.0%)^a^	.021
Stroke, *n* (%)	2 (5.4%)	3 (4.0%)	4 (12.9%)	.272
PAOD, *n* (%)	0 (0.0%)	1 (1.3%)	1 (3.2%)	.453
HFrEF, *n* (%)	22 (59.5%)	42 (56.0%)	15 (48.4%)	.646
Laboratory findings				
WBC count (x10^3^/L)	6.3 ± 1.4	6.8 ± 1.7	6.3 ± 1.6	.223
Hemoglobin (g/dL)	13.6 ± 1.5	13.4 ± 1.6	12.3 ± 1.6*	.001
Platelet count (x10^3^/L)	200.9 ± 44.4	211.3 ± 50.0	197.3 ± 44.2	.305
eGFR (mL/min/1.73 m^2^)	75.6 ± 17.2	70.0 ± 20.5	56.1 ± 23.7*	<.001
NT‐proBNP (pg/mL)	416.3 ± 485.4	776.3 ± 1557.8	1861.5 ± 3775.3	.017
Echocardiographic findings				
LVEDD (mm)	51.6 ± 8.6	52.6 ± 9.1	51.6 ± 8.8	.807
LVEF (%)	49.2 ± 16.0	49.5 ± 16.9	53.3 ± 15.9	.511
LVMI (g/m^2^)	113.2 ± 31.0	112.2 ± 31.8	109.5 ± 30.0	.885
LAVI (ml/m^2^)	35.4 ± 13.1	41.3 ± 24.3	43.4 ± 17.0	.251
E/E'	12.2 ± 4.9	14.2 ± 7.2	13.5 ± 3.9	.312

Abbreviations: BMI, body mass index; CABG, coronary artery bypass graft; CAOD, coronary artery obstructive disease; eGFR, estimated glomerular filtration rate; HFpEF, heart failure with preserved ejection fraction; HFrEF, heart failure with reduced ejection fraction; ICMP, ischemic cardiomyopathy; LAVI, left atrial volume index; LVDEE, left ventricular end diastolic diameter; LVEF, left ventricular ejection fraction; LVMI, left ventricular mass index; MI, myocardial infarction; NICMP, nonischemic cardiomyopathy; NT‐proBNP, N‐terminal pro b‐type natriuretic peptide; PAOD, peripheral artery obstructive disease; PCI, percutaneous coronary intervention; WBC, white blood cell.

* *p*‐value < .05 (*t*‐test for “Frail” vs. “Robust or Prefrail”).

^a^ *p*‐value < .05 (Chi‐squared test for “Frail” vs. “Robust or Prefrail”).

### 3.2 Physical fitness parameters and BIA findings

Table 2 shows the results of CPET, 6MWT, muscular fitness tests, and BIA. Frail patients had a significantly lower peak VO_2,_ and respiratory exercise ratio on CPET, and their 6MWT scores were significantly lower than other groups. Compared with robust patients, both peak VO_2_ and 6MWT were significantly decreased in prefrail and frail patients (Supporting Information: Figure 2A and 2B). The 6MWT score was significantly lower in frail patients than in prefrail patients (Supporting Information: Figure 2B). However, in the muscular fitness tests, there was no difference in MVIC, MP, or handgrip strength between robust, prefrail, and frail patients (Supporting Information: Figure 2C **–** 2F). These results were similar for each parameter according to the K‐FRAIL scores (Supporting Information: Figure 3). In the BIA, body fat mass and visceral fat area were higher in frail patients.

**Table 2:** Parameters of aerobic capacity tests, muscle fitness measurement, and bioelectrical impedance analysis.

	Robust (*n* = 37)	Prefrail (*n* = 75)	Frail (*n* = 31)	*p*‐Value
Cardiopulmonary exercise test				
Peak VO_2_ (mL/kg/min)	22.8 ± 5.0	19.3 ± 4.6	16.9 ± 4.7*	<.001
Age‐predicted aerobic capacity (%)	97.9 ± 24.1	83.1 ± 21.7	83.1 ± 26.2	.008
RER at peak	1.00 ± 0.10	0.98 ± 0.10	0.98 ± 0.10	.555
VE/VCO_2_ slope	30.3 ± 4.5	33.3 ± 7.9	37.6 ± 10.1*	.640
6MWT (m)	458.4 ± 68.2	404.5 ± 92.3	311.2 ± 120.5*	<.001
Muscle fitness				
Handgrip strength, extension (N)	23.8 ± 6.4	22.7 ± 7.3	21.9 ± 5.9	.512
Handgrip strength, flexion (N)	22.6 ± 6.1	20.9 ± 7.1	19.9 ± 5.8	.253
MVIC (N)	298.9 ± 81.7	289.0 ± 114.0	294.9 ± 149.3	.906
MP (W)	83.4 ± 34.5	78.3 ± 46.9	73.3 ± 42.5	.631
Bioelectrical impedance analysis				
Soft lean mass, kg	41.4 ± 7.1	39.5 ± 7.6	41.4 ± 9.7	.449
Fat‐free mass, kg	43.9 ± 7.5	41.9 ± 8.0	43.9 ± 10.1	.447
Skeletal muscle mass, kg	23.8 ± 4.5	22.6 ± 4.8	23.6 ± 6.1	.455
Body fat mass, kg	19.8 ± 6.4	20.1 ± 5.8	24.4 ± 9.8*	.022
Percent body fat, %	30.7 ± 7.6	32.4 ± 8.3	35.1 ± 8.2	.138
Visceral fat area, cm^2^	95.5 ± 37.1	102.5 ± 37.6	130.2 ± 63.1*	.011

Abbreviations: 6MWT, 6‐min walk test; MP, muscle power; MVIC, maximum voluntary isomeric contraction; RER, respiratory exercise ratio; VCO_2_, carbon dioxide production; VE, ventilatory equivalents; VO_2_, oxygen uptake.

* *p*‐value < .05 (*t*‐test for “Frail” vs. “Robust or Prefrail”).

### 3.3 Association between aerobic exercise capacity and K‐FRAIL score

Multivariate analysis revealed that peak VO_2_ (*B* ± standard error = −0.070 ± 0.022, *β* = −.311, *p* = .002) and 6MWT score (*B* ± standard error = −0.004 ± 0.001, *β* = −.382, *p* < .001) were significantly correlated with the K‐FRAIL score (Table 3). However, MVIC, MP, and handgrip strength were not independently correlated with the K‐FRAIL score (Supporting Information: Tables 1 **–** 4).

**Table 3:** Multivariate analysis to identify independent predictors of K‐FRAIL score.

	Univariate analysis			Multivariate analysis			Multivariate analysis		
Variables	*B* ± SE	*β*	*p*‐Value	*B* ± SE	*β*	*p*‐Value	*B* ± SE	*β*	*p*‐Value
Age, years	0.017 ± 0.006	.231	.006	0.017 ± 0.022	.081	.423	−0.008 ± 0.021	−.040	.683
Sex, female	−0.081 ± 0.069	−.098	.244	−0.192 ± 0.248	−.083	.439	0.129 ± 0.214	.055	.553
Hemoglobin, g/dL	−0.076 ± 0.020	−.301	<.001	−0.035 ± 0.073	−.047	.633	−0.083 ± 0.064	−.116	.199
eGFR, ml/min/1.73m^2^	−0.006 ± 0.002	−.304	<.001	−0.005 ± 0.005	−.082	.405	−0.007 ± 0.005	−.134	.132
LVEF, %	0.009 ± 0.006	.119	.164	0.003 ± 0.008	.049	.645	0.006 ± 0.007	.090	.346
Peak VO_2_, mL/kg/min	−0.020 ± 0.007	−.270	.003	−0.070 ± 0.022	−.311	.002			
6MWT, m	−0.002 ± 0.000	−.429	<.001				−0.004 ± 0.001	−.384	<.001

Abbreviations: 6MWT, 6‐min walk test; eGFR, estimated glomerular filtration rate; LVEF, left ventricular ejection fraction; SE, standard error; VO_2_, oxygen uptake.

### 3.4 Cut‐off values of peak VO_2_ and 6MWT for frailty according to the K‐FRAIL scale

**Figure 2:**
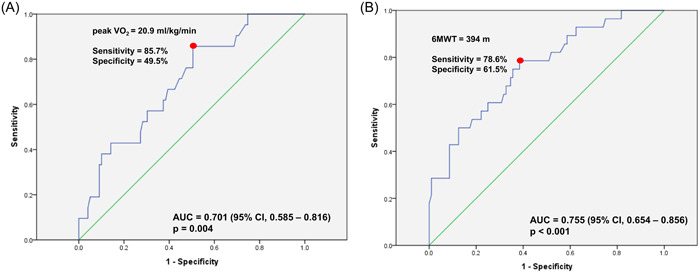
ROC curve analysis to predict frailty by K‐FRAIL Scale (A) ROC curve analysis for peak VO_2_ to predict frailty by K‐FRAIL scale. (B) ROC curve analysis for 6MWT to predict frailty by K‐FRAIL scale. 6MWT, 6‐min walk test; AUC, area under curve; CI, confidence interval; ROC, receiver‐operating characteristic; VO_2_, oxygen uptake.

The ROC curves showed that the cut‐off values of peak VO_2_ and 6MWT for frailty based on the K‐FRAIL scale were 20.9 mL/kg/min and 394 m, respectively (Figures 2A,B). The cut‐off values of peak VO_2_ and 6MWT had a sensitivity and specificity of 85.7% and 49.5%, and 78.6% and 61.5%, respectively. MVIC, MP, and handgrip strength showed no significant ROC curves for frailty according to the K‐FRAIL scale (Supporting Information: Figure 4).

In the multivariable Cox proportional hazard model for the cut‐off value (Table 4), peak VO_2_ < 20.9 mL/kg/min (hazard ratio [HR], 5.08; 95% confidence interval [CI], 1.25–20.6, *p* = .023) and 6MWT < 394 m (HR, 3.99; 95% CI, 1.25–12.8, *p* = .020) were independent predictors of frailty according to the K‐FRAIL scale.

**Table 4:** Multivariable Cox proportional hazard regression analyses for frail status by the K‐FRAIL scale.

Variables	HR (95% CI)	*p*‐Value	Variables	HR (95% CI)	*p*‐Value
Age	1.050 (0.938–1.174)	.397	Age	1.002 (0.904–1.11)	.975
Female	0.352 (0.095–1.299)	.117	Female	0.920 (0.308–2.748)	.881
Hemoglobin	0.798 (0.535–1.190)	.798	Hemoglobin	0.740 (0.528–1.037)	.081
eGFR	0.992 (0.963–1.21)	.992	eGFR	0.974 (0.950–0.999)	.044
LVEF	0.996 (0.957–1.037)	.847	LVEF	1.006 (0.971–1.042)	1.006
Peak VO_2_ < 20.9 mL/kg/min	5.359 (1.300–22.092)	.020			
			6MWT < 394 m	4.058 (1.224–13.458)	.022

Abbreviations: 6MWT, 6‐min walk test; CI, confidence interval; eGFR, estimated glomerular filtration rate; HR, hazard ratio; LVEF, left ventricular ejection fraction; VO_2_, oxygen uptake

## 4 DISCUSSION

In this study, we verified the extent to which the K‐FRAIL scale reflects differences in physical characteristics and physical fitness in older patients with ambulatory HF. Patients classified as frail by the K‐FRAIL scale tended to have a higher BMI than those who were not frail, and this difference was mainly due to the difference in fat mass. Regarding handgrip and lower extremity muscular fitness, there was no difference between frail patients and robust or prefrail patients. However, the 6MWT and peak VO_2_ gradually decreased with an increase in the degree of frailty. Therefore, we can conclude that the K‐FRAIL scale correlates better with aerobic exercise capacity than with muscular fitness in older ambulatory HF patients.

Frailty is often triggered by poor health conditions related to the disease; however, frailty itself is a major factor that exacerbates the disease. Therefore, early screening for frailty and appropriate intervention can improve frailty‐related health outcomes.^18^ Multiple instruments for screening and evaluating frailty, which can be divided into self‐reported, performance‐based, and a combination of both, have been developed.^19^ Compared with the self‐reported type, a performance‐based instrument has advantages such as high sensitivity to changes or differences in activity and nonresponse, high accuracy and validity of response, and low risk of bias as answers would not depend on the respondent's mood or perception. However, it is limited in that it requires special equipment, a location for measurement, trained inspectors, and sufficient time for measurement.^20^, ^21^, ^22^

K‐FRAIL, the Korean translation of the frailty scale by Morley et al.,^13^ is a frailty screening instrument in the form of a self‐reported questionnaire that can be easily completed by patients or guardians with the help of a nurse.^10^ In Korean older patients, the K‐FRAIL scale showed a good correlation with the multidimensional frailty index calculated using more than 70 variables obtained from the comprehensive geriatric assessment.^10^ A previous study showed that the resistance and ambulation items of the K‐FRAIL scale are closely related to physical performance, such as activities of daily living or instrumental activities of daily living.^10^ In our study, K‐FRAIL showed a correlation with aerobic capacity, but not musculoskeletal strength/capacity, which was evaluated objectively by performance‐based tests.^10^

The handgrip test measures muscular fitness in older patients, and weakness, one of the criteria for the physical frailty phenotype, is assessed by this test.^1^, ^23^ The handgrip strength is also an independent predictor of prognosis in patients with HF.^24^ The use of the handgrip test was favored over a multidimensional assessment because of the patients' minimal tolerance for physical exertion and disease‐related deconditioning, which are common in patients with HF.^25^ However, in our study, the degree of frailty did not significantly affect handgrip strength. As handgrip strength is closely related to muscle mass,^26^ the nonsignificant difference between groups may be explained by the nonsignificant difference in the muscle mass between robust, prefrail, and frail patients.

Muscles of the lower extremities are mainly responsible for mobility in relation to daily living. Lower extremity muscle mass, strength, and function are closely associated with frailty.^27^, ^28^ In particular, patients with HF experience easy fatigability of the lower extremities,^29^ which leads to limitations in exercise and mobility. In addition, knee extensor muscle power in patients with HFrEF is an independent predictor of HF rehospitalization.^17^ However, similar to handgrip strength, muscular strength and power of the lower extremities did not significantly differ between the degrees of the frailty of the K‐FRAIL scale.

In contrast, the K‐FRAIL scale showed a good correlation with the aerobic functional capacity test results. The measured VO_2_ during maximal symptom‐limited CPET is an index that most objectively evaluates aerobic exercise capacity. Peak VO_2_ is the highest value of VO_2_ attained during high‐intensity exercise and is an important predictor in patients with HF.^30^ Peak VO_2_ was reported to correlate with the evaluation index of sarcopenia and frailty in older patients.^31^ In our study, peak VO_2_ correlated well with the K‐FRAIL scale even after adjustment for LVEF and was significantly associated with the frailty status.

Similarly, the 6MWT was correlated with frailty. While CPET requires complex protocols, special instruments, and trained examiners, the 6MWT can be applied very easily and safely. In patients with HF, the results of the 6MWT within 300 m is highly related to frailty.^32^ In our study, the 6MWT results gradually decreased according to the degree of frailty and showed a significant association with frailty status even after adjusting for several confounding variables, including LVEF.

Both peak VO_2_ and the 6MWT were found to be reliable test methods for screening frailty in this study. However, in symptom‐limited CPET, peak VO_2_ is an index that can be obtained from maximal exercise; therefore, it is somewhat difficult to measure physical performance in frail older patients. In contrast, the 6MWT is a submaximal exercise test that allows the examinee to rest during the test and can be used more easily and conveniently than CPET in older patients with HF. The 6MWT is a good measure of physical performance because it correlates with muscle fitness and aerobic capacity.^33^

### 4.1 Study limitations

First, frailty is a clinical syndrome, that is, difficult to define with multi‐domain aspects, and there is no gold standard for its diagnosis. However, this is a problem in all frailty studies. The main purpose of this study was to resolve the question of how much K‐FRAIL can reflect the physical aspect of frailty; therefore, the concern for the diagnosis of frailty may not be a problem. Second, this study did not include patients with severe and vulnerable HF with reduced mobility as it is difficult to perform objective single performance‐based tests in these patients.

## 5 CONCLUSIONS

Physical performance differs according to the degree of frailty in older HF patients. Peak VO_2_ and 6MWT correlated with the K‐FRAIL scale better than muscular fitness and are good independent predictors of frailty.

## CONFLICT OF INTERESTS STATEMENT

The authors declare no conflict of interest.

## Supporting information

Supporting information.

## Data Availability

The data that support the findings of this study are available on request from the corresponding author.
