# Supplementary material for: Association between frailty and physical performance in older patients with heart failure
Source: Clin Cardiol. 2023 Sep 7;46(12):1530–7. doi: 10.1002/clc.24142 (PMC10716321; doi:10.1002/clc.24142)
Supplement: Supplementary file 1 — Supporting information. [file CLC-46-1530-s001.docx]

**The Association between Frailty and Physical Performance in Elderly Patients with Heart Failure**

**- SUPPLEMENTARY MATERIALS -**

**SUPPLEMENTARY FIGURE LEGENDS**

**Supplementary Figure 1. Frailty assessments.**

**Supplementary Figure 2. Aerobic exercise capacity according to K-FRAIL scale.**

**Supplementary Figure 3. Aerobic exercise capacity according to K-FRAIL score.**

**Supplementary Figure 4. ROC Curve Analysis to Predict Frailty by K-Frail Scale.**

**Supplementary Table 1. Multivariate Analysis to Identify Independent Predictors of K-Frail Scale (MVIC).**

**Supplementary Table 2. Multivariate Analysis to Identify Independent Predictors of K-Frail Scale (MP).**

**Supplementary Table 3. Multivariate Analysis to Identify Independent Predictors of K-Frail Scale (Hand grip strength, extension).**

**Supplementary Table 4. Multivariate Analysis to Identify Independent Predictors of K-Frail Scale (Hand grip strength, flexion).**

**Supplementary Figure 1. Frailty assessments.** FRAIL scale items in African American Health.


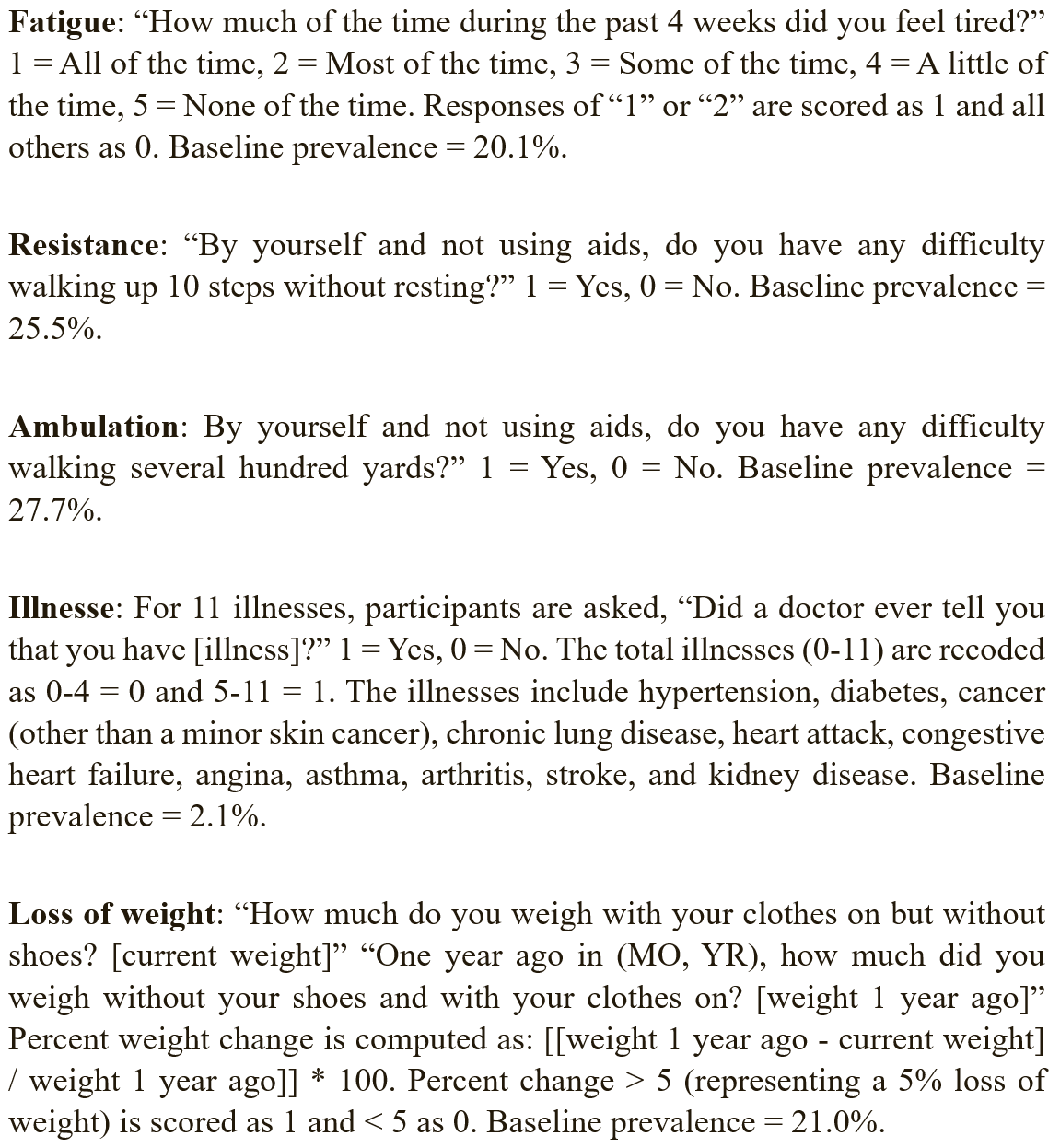


**Supplementary Figure 2. Aerobic exercise capacity according to K-FRAIL scale.**

(A) Peak VO_2_, (B) 6MWT, (C) MVIC, (D) MP, (E) Hand grip strength, extension, (F) Hand grip strength, flexion. (*p < 0.05, **p < 0.01, *** p < 0.001)

6MWT, 6-minute walk test; MP, muscle power; MVIC, maximum voluntary isomeric contraction; VO2, oxygen uptake.


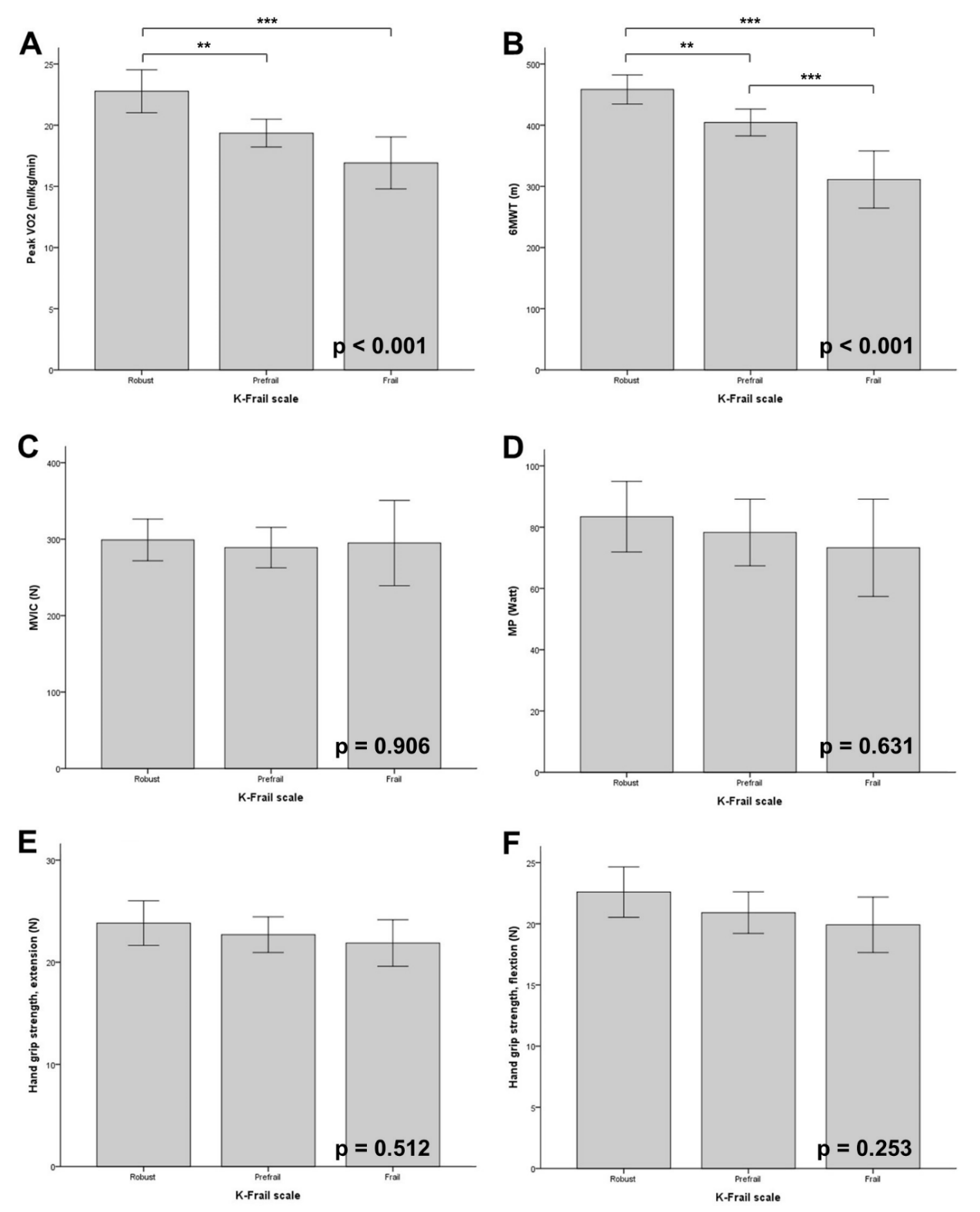


**Supplementary Figure 3. Aerobic exercise capacity according to K-FRAIL score.**

(A) Peak VO_2_, (B) 6MWT, (C) MVIC, (D) MP, (E) Hand grip strength, extension, (F) Hand grip strength, flexion. (*p < 0.05, **p < 0.01, *** p < 0.001)

6MWT, 6-minute walk test; MP, muscle power; MVIC, maximum voluntary isomeric contraction; VO2, oxygen uptake.


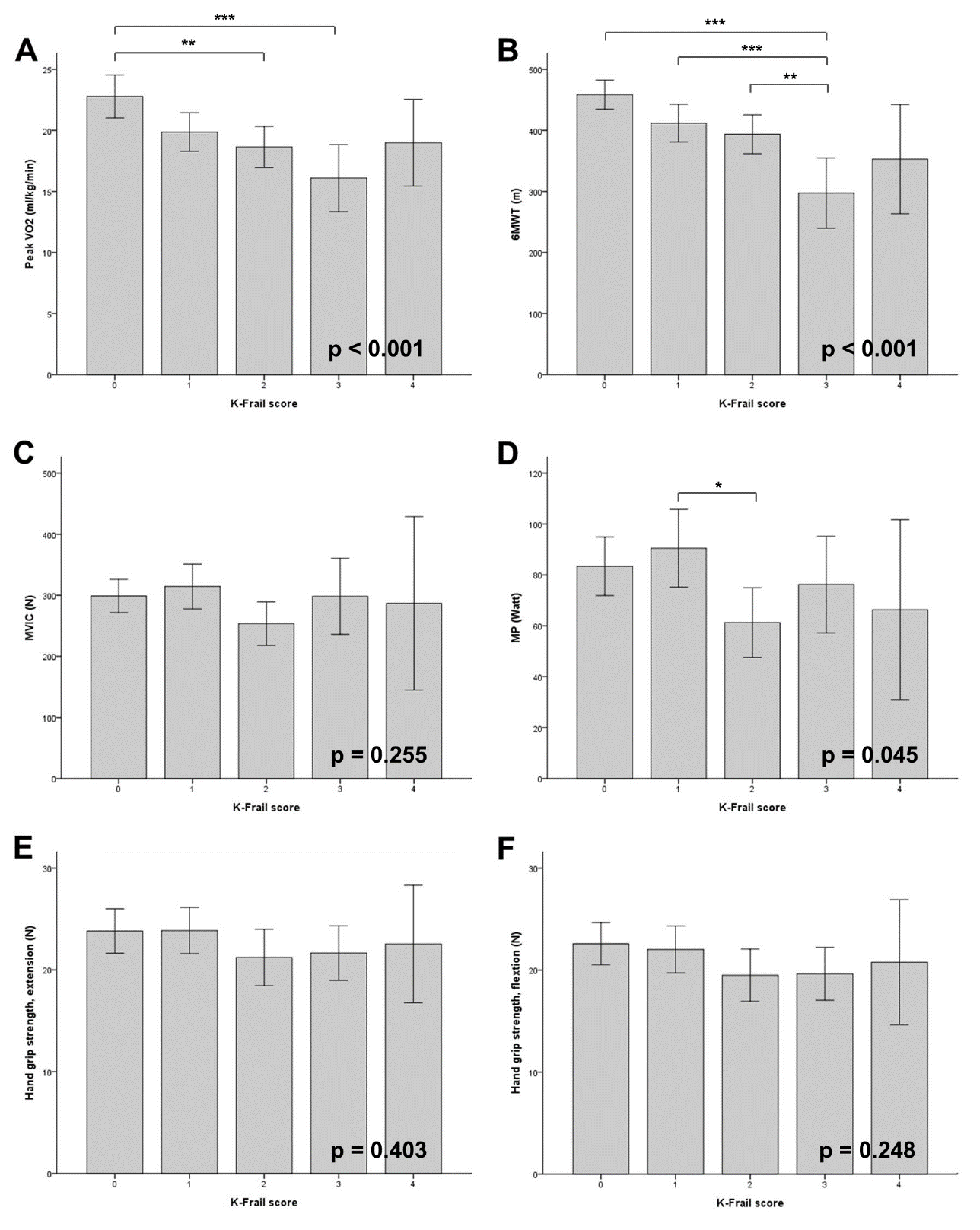


**Supplementary Figure 4. ROC Curve Analysis to Predict Frailty by K-Frail Scale.**

ROC curve analysis for (A) MVIC, (B) MP, (C) hand grip strength (extension), and (D) hand grip strength (flexion) to predict frailty by K-Frail scale.

AUC, area under curve; CI, confidence interval; MP, muscle power; MVIC, maximum voluntary isomeric contraction; ROC, receiver-operating characteristic.


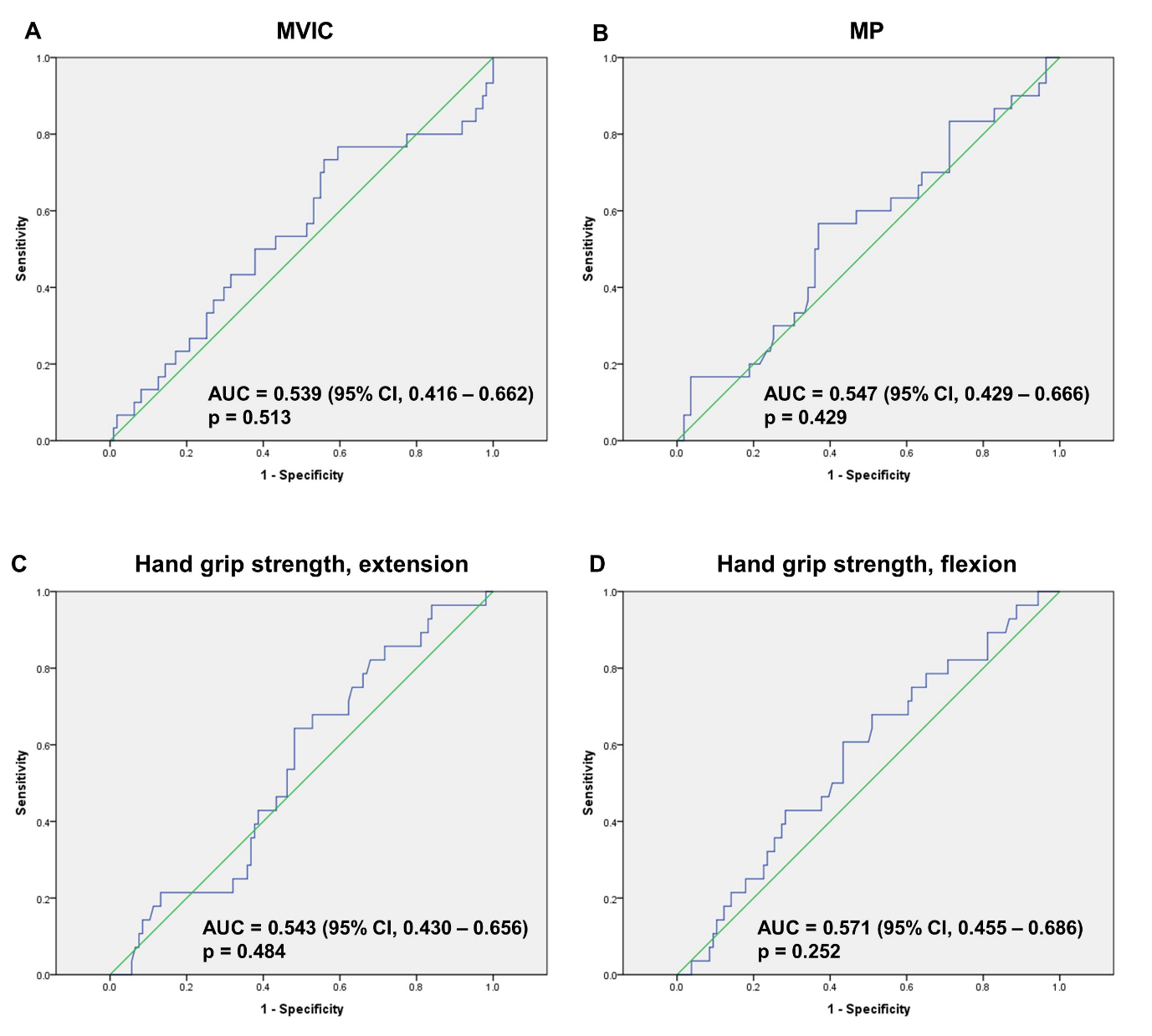


**Supplementary Table 1. Multivariate Analysis to Identify Independent Predictors of K-Frail Scale (MVIC).**

|  | Univariate analysis | | | Multivariate analysis | | |
| --- | --- | --- | --- | --- | --- | --- |
| Variables | B ± SE | β | *P*-value | B ± SE | β | *P*-value |
| Age, years | 0.017±0.006 | 0.231 | 0.006 | 0.034±0.020 | 0.158 | 0.090 |
| Sex, female | -0.081±0.069 | -0.098 | 0.244 | 0.001±0.238 | 0.001 | 0.995 |
| Hemoglobin, g/dL | -0.076±0.020 | -0.301 | <0.001 | -0.102±0.066 | -0.141 | 0.125 |
| eGFR, ml/min/1.73m^2^ | -0.006±0.002 | -0.304 | <0.001 | -0.013±0.005 | -0.232 | 0.008 |
| LVEF, % | 0.009±0.006 | 0.119 | 0.164 | 0.007±0.007 | 0.094 | 0.332 |
| MVIC, N | -0.001±0.001 | -0.071 | 0.405 | 0.000±0.001 | 0.027 | 0.762 |

eGFR, estimated glomerular filtration rate; LVEF, left ventricular ejection fraction; MVIC, maximum voluntary isomeric contraction; SE, standard error.

**Supplementary Table 2. Multivariate Analysis to Identify Independent Predictors of K-Frail Scale (MP).**

|  | Univariate analysis | | | Multivariate analysis | | |
| --- | --- | --- | --- | --- | --- | --- |
| Variables | B ± SE | β | *P*-value | B ± SE | β | *P*-value |
| Age, years | 0.017±0.006 | 0.231 | 0.006 | 0.032±0.020 | 0.148 | 0.114 |
| Sex, female | -0.081±0.069 | -0.098 | 0.244 | 0.049±0.239 | 0.020 | 0.838 |
| Hemoglobin, g/dL | -0.076±0.020 | -0.301 | <0.001 | -0.097±0.066 | -0.135 | 0.145 |
| eGFR, ml/min/1.73m^2^ | -0.006±0.002 | -0.304 | <0.001 | -0.013±0.005 | -0.230 | 0.009 |
| LVEF, % | 0.009±0.006 | 0.119 | 0.164 | 0.007±0.007 | 0.099 | 0.303 |
| MP, Watt | -0.004±0.002 | -0.157 | 0.063 | -0.001±0.003 | -0.028 | 0.760 |

eGFR, estimated glomerular filtration rate; LVEF, left ventricular ejection fraction; MP, muscle power; SE, standard error.

**Supplementary Table 3. Multivariate Analysis to Identify Independent Predictors of K-Frail Scale (Hand grip strength, extension).**

|  | Univariate analysis | | | Multivariate analysis | | |
| --- | --- | --- | --- | --- | --- | --- |
| Variables | B ± SE | β | *P*-value | B ± SE | β | *P*-value |
| Age, years | 0.017±0.006 | 0.231 | 0.006 | 0.031±0.021 | 0.143 | 0.138 |
| Sex, female | -0.081±0.069 | -0.098 | 0.244 | -0.150±0.288 | -0.063 | 0.603 |
| Hemoglobin, g/dL | -0.076±0.020 | -0.301 | <0.001 | -0.100±0.068 | -0.139 | 0.145 |
| eGFR, ml/min/1.73m^2^ | -0.006±0.002 | -0.304 | <0.001 | -0.011±0.005 | -0.203 | 0.025 |
| LVEF, % | 0.009±0.006 | 0.119 | 0.164 | 0.008±0.007 | 0.115 | 0.239 |
| Hand grip strength, extension, N | -0.023±0.015 | -0.131 | 0.132 | 0.010±0.020 | 0.058 | 0.622 |

eGFR, estimated glomerular filtration rate; LVEF, left ventricular ejection fraction; SE, standard error.

**Supplementary Table 4. Multivariate Analysis to Identify Independent Predictors of K-Frail Scale (Hand grip strength, flexion).**

|  | Univariate analysis | | | Multivariate analysis | | |
| --- | --- | --- | --- | --- | --- | --- |
| Variables | B ± SE | β | *P*-value | B ± SE | β | *P*-value |
| Age, years | 0.017±0.006 | 0.231 | 0.006 | 0.030±0.021 | 0.141 | 0.147 |
| Sex, female | -0.081±0.069 | -0.098 | 0.244 | -0.123±0.287 | -0.052 | 0.669 |
| Hemoglobin, g/dL | -0.076±0.020 | -0.301 | <0.001 | -0.098±0.068 | -0.136 | 0.151 |
| eGFR, ml/min/1.73m^2^ | -0.006±0.002 | -0.304 | <0.001 | -0.011±0.005 | -0.206 | 0.024 |
| LVEF, % | 0.009±0.006 | 0.119 | 0.164 | 0.008±0.007 | 0.116 | 0.236 |
| Hand grip strength, flexion, N | -0.030±0.015 | -0.166 | 0.055 | 0.007±0.021 | 0.041 | 0.731 |

eGFR, estimated glomerular filtration rate; LVEF, left ventricular ejection fraction; SE, standard error.
